# An explorative study on proteomic analyses related to inflammation and pain in children with juvenile idiopathic arthritis

**DOI:** 10.1186/s12887-023-04181-0

**Published:** 2023-07-15

**Authors:** Andreas Elfving, Arja Harila-Saari, Ludwig Nilsson, Lillemor Berntson

**Affiliations:** Department of Women’s and Children’s Health, Uppsala University, 75185 Uppsala, Sweden

**Keywords:** Arthritis, juvenile, Proteomics, Pain, GDNF

## Abstract

**Background:**

Our aim was attempting to find proteins involved in the pain process and correlating with pain but not degree of inflammation in children with juvenile idiopathic arthritis (JIA), using a proteomics panel.

**Methods:**

A total of 87 plasma samples were collected from 51 children with JIA (51 at diagnosis in a higher disease activity state, 18 at follow-up in a lower disease activity state) and 18 healthy controls. Relative levels of 92 proteins related to a wide range of biological processes in inflammation were obtained using a proximity extension assay panel. Comparisons between children with and without JIA, in different disease categories, by juvenile disease activity score (JADAS27) and degree of pain on a visual analogue scale (VAS), were performed using parametric and non-parametric statistical methods.

**Results:**

Nineteen proteins involved in arthritic inflammation, such as interleukin 6 (IL-6) and S100 protein A12, were higher in patients with JIA than controls, seven decreased significantly during treatment, and 18 correlated significantly with JADAS27. Three proteins correlated with pain VAS scores in unadjusted analyses: the glial cell line-derived neurotrophic factor (GDNF), transforming growth factor beta, and IL-18R1. Levels of GDNF correlated significantly with pain VAS scores but not with JADAS27.

**Conclusions:**

Plasma levels of 18 of 92 tested proteins correlated with degree of disease activity. Levels of three proteins correlated with pain, and levels of one, GDNF, originating from neural cells, correlated with pain without correlating with inflammatory degree, suggesting that it may play a role in pain in JIA. Further studies in larger cohorts are warranted.

**Supplementary Information:**

The online version contains supplementary material available at 10.1186/s12887-023-04181-0.

## Background

Juvenile idiopathic arthritis (JIA) is the most common rheumatic disease during childhood. JIA is an umbrella term used to describe a heterogeneous autoimmune disease comprising seven categories, all presenting with an arthritis for at least six weeks with onset before 16 years of age [1].

Many cells and proteins are involved in the inflammatory and pain processes in JIA. Some proteins are well-studied in JIA-related inflammation, e.g., tumor necrosis factor alpha (TNFα), IL-1, IL-6, and the S100 proteins. The IL-6 is a pleiotropic cytokine that mediates acute phase reactions and is produced in a number of different cells. The S100 proteins, S100A12 and S100A8/9, originate from neutrophils and monocytes and are studied for disease activity in JIA [2, 3]. They have also been studied as possible indicators of risk of reactivation, without convincing results [4].

In addition to control of inflammation, a key goal of JIA treatment is to reduce pain, yet assessment of pain has not been incorporated in composite outcome measures for JIA [5]. The mechanism of pain in JIA is not fully understood and the experience of pain is related to many factors, for example disease activity, psychosocial situation, comorbidity, and quality of sleep. Many components are involved in the pain signaling system and so far there are no known specific biomarkers for pain in JIA. When monitoring disease activity, the focus has been on identifying biomarkers involved in inflammation. Inflammatory biomarkers in arthritis are often also described to relate to degree of pain, but children with JIA commonly report pain even when there are no signs of inflammatory activity [6, 7].

Studies analyzing multiple proteins/cytokines in JIA are rare and focused mainly on systemic JIA [8, 9]. Cytokine patterns most likely differ between the six disease categories, excepting the systemic category, but we have no knowledge of the extent to which they do so. In a study by Brescia et al*.*, significant differences were observed in levels of cytokines associated with inflammation in synovial fluid samples from children with JIA compared to controls [10]. One study using liquid chromatography/mass spectrometry analyzed the serum proteome in 15 children with JIA and found 14 inflammatory and non-inflammatory proteins correlating significantly with clinical pain severity [11].

We used the inflammatory proteomics panel by Olink [12] to analyze concentrations of 92 proteins involved in a wide range of biological processes in inflammation and related the results to disease activity and pain in children with JIA.

## Methods

### Patients

In this clinical explorative study, we prospectively included 51 children at the Unit of Pediatric Rheumatology, Uppsala University Hospital, between 2017 and 2019. The participants were diagnosed and classified with JIA based on the International League of Association for Rheumatology criteria [1]. Children with systemic JIA were excluded because of its different pathophysiology compared with other disease categories. The 51 patients with JIA were examined in a highly active disease state, directly after confirmed diagnosis but before receiving any other treatment than non-steroid anti-inflammatory drugs. Eighteen of the 51 children, randomly chosen, were also examined at follow-up in a lower disease activity state, during (or after) medical treatment. The numbers of children in different disease categories, excluding the systemic category, were largely representative of disease occurrence in the general population.

### Healthy children

We also included pre-operatively collected blood samples of 18 healthy children admitted for minor surgery. Exclusion criteria were medication for any disease, presence of any inflammatory disease, diabetes or any atopic disease with continuous medication or special diet because of intolerance. Blood samples were drawn before surgery.

### Methods

In addition to blood sampling, study visits included assessment of the juvenile disease activity score (JADAS27) for monitoring disease activity in the children with JIA and scoring of pain on a visual analogue scale (VAS) (0–10 cm). Eighteen of the 51 children with JIA had a follow-up study visit. The JADAS27 comprises a joint count (0–27 active joints), patient-reported global assessment of well-being on a VAS (0 – 10 cm) (assessed by a parent if the child is ≤ 9 years old), physician’s global assessment of disease activity on VAS (0 –10 cm) and normalized erythrocyte sedimentation rate ((E-SR in mm/h)—20)/10) to a scale (0–10) with a maximum total score of 57 [13].

Blood samples were centrifuged, aliquoted, and frozen in -70 °C within 3.5 h. In addition to analyses of E-SR, C-reactive protein, and leukocytes in children with JIA, we used an inflammatory proteomics panel, performed by Olink (Sweden) in all 87 samples. The method is based on a well-characterized nucleic acid proximity-based assay using antibodies, called Proximity Extension Assay, with good performance in plasma samples (http://www.olink.com/). Results are presented as normal protein expression (NPX) values, which are relative quantification values and not exact levels of the protein biomarkers.

### Statistical methods

Demographic data were given using descriptive statistics with interquartile range (IQR) and median (Md). The Mann–Whitney U test was used for comparisons of two groups. For differences in clinical features before and during treatment, the related samples Wilcoxon signed-rank test was used (Additional Table 1). Independent samples T-test was performed for comparison of the 19 significant biomarkers and the difference between children with JIA and controls (Additional Table 3). Adjustment for age was performed with linear regression in Additional Tables 4 and 5. Adjustment for age and gender was performed in Spearman’s rank correlation analyses between NPX-levels and pain VAS in the 51 children with JIA (Table 3). A paired samples T-test was used for comparison of paired samples in 18 children with follow-up data. Spearman’s rank correlation and partial correlation (adjusted for age and gender) analyses were used for the evaluation of associations between biomarker levels and JADAS27 as well as pain VAS scores. All results are presented both with and without adjustment for multiple testing, using the Benjamini–Hochberg method [14]. All tests were considered significant if *p* < 0.05. Analyses were performed using the Statistical Package for Social Sciences version 28 (SPSS Inc., Illinois, USA).

**Table 1:** Demographic data for 51 children with juvenile idiopathic arthritis (JIA) and 18 healthy children

	**Children with JIA**		**Healthy children**
	**Total cohort at diagnosis** ***n* = 51**	**Follow-up cohort** ***n* = 18**	***n* = 18**
Age at inclusion, Md (IQR)	10.4 (5.6 – 15.2) *	9.4 (5.0 – 13.6)	5.6 (2.2 – 9.4) *
Age at onset, Md (IQR)	8.8 (3.2 – 13.5)	6.2 (3.0 – 11.5)	-
Sex (F/M), n (%)	34/17 (66.7)	12/6 (66.7)	3/15 (16.7)
Disease duration at inclusion, years, Md (IQR) Disease duration at follow-up, years, Md (IQR)	0.6 (0.2 – 2.7) -	- 1.2 (0.7 – 2.6)^a^	- -
**ILAR categories, course type, n (%)**			
Oligoarticular persistent	25 (49)	7 (38.9)	-
Enthesitis-related arthritis	9 (17.6)	3 (16.7)	-
Polyarticular RF negative	7 (13.7)	3 (16.7)	-
Juvenile psoriatic arthritis	4 (7.8)	2 (11.1)	-
Oligoarticular extended	3 (5.9)	2 (11.1)	-
Polyarticular RF positive	3 (5.9)	1 (5.6)	-
**Medical treatment at follow-up**			
Methotrexate	-	9	-
Etanercept	-	2	-
Etanercept + Methotrexate	-	5	-
No medical treatment	-	2	-

*ILAR* International League of Associations for Rheumatology (ILAR) criteria

*Md* Median, *IQR* Interquartile range, *F* Female, *M* Male, *Comparison of age at inclusion between the total cohort and healthy children using Mann Whitney U-test, *p*-value = 0.007

^a^Median time from visit at baseline and visit at follow up in 18 children, 7 (IQR 5.2–8.8) months

## Results

**Fig. 1:**
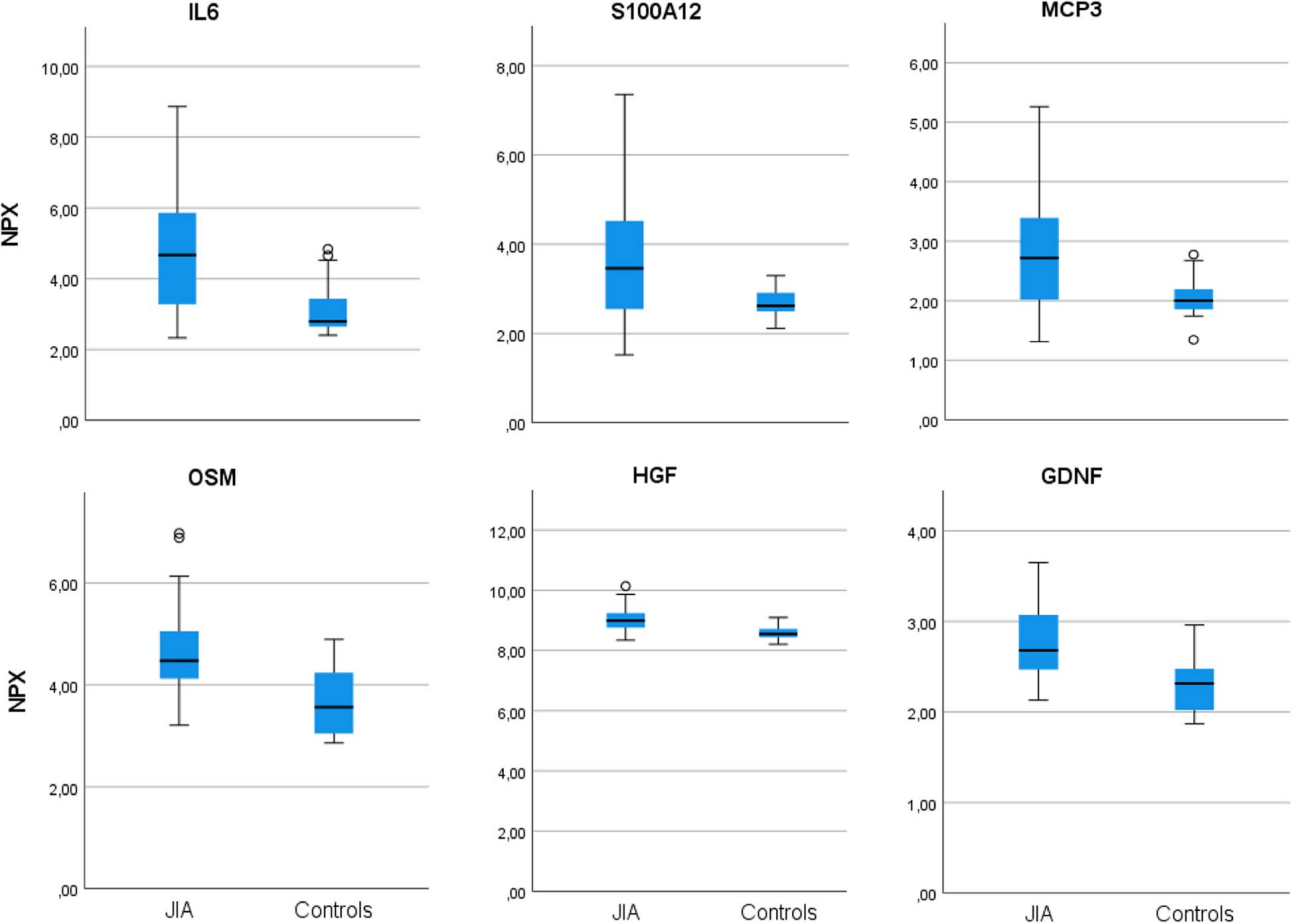
Boxplots presenting normal protein expression (NPX) values, adjusted for multiple analyses, of the six proteins differing most significantly between 51 children with JIA and 18 controls, from a panel of 92 inflammatory proteins. The *p*-value was < 0.001 in all six analyses, according to independent samples T-test

Demographic data of study participants is presented in Table 1. The 51 children with JIA had a median age at inclusion of 10.4 years. Six of the seven categories of the International League of Associations for Rheumatology were represented. Eighteen of the 51 children had follow-up examinations at a Md of 7 (IQR 5.2–8.8) months. The medical treatment at the time of follow-up examination is presented, but not the medical treatments given between baseline and follow-up. Clinical and conventional laboratory variables are presented in Additional Table 1. During follow-up, JADAS27 decreased from Md 11.3 (IQR 7.4–18.9) to 2.6 (IQR 0.3–3.7), *p* < 0.001. The E-SR decreased in the 18 children from Md 24.0 (IQR 9.5–41.5) to Md 7.5 (IQR 2.0–10.8) mm, *p* < 0.001. Levels of pain and JADAS27 in the different disease categories are shown in Additional Table 2. Figure 1 shows the six proteins differing most significantly in NPX values, adjusted for multiple testing, between the 51 children with JIA in an active inflammatory state and controls: IL-6, S100A12, monocyte chemotactic protein-3 (MCP-3), oncostatin M (OSM), hepatocyte growth factor (HGF) and glial cell line derived neurotrophic factor (GDNF). Additional Table 3 presents all 19 proteins differing significantly in NPX values between the 51 children with JIA in an active inflammatory state and controls. The results were significant even after adjustment for multiple testing. The 18 healthy children had a lower age at inclusion than the 51 children with JIA, but after adjusting for age, using linear regression analyses, there was still a significant difference between groups (Additional Table 4). Because of a skewed gender distribution in children with JIA compared to controls with only three girls in the control group, we performed an analysis using linear regression in the two groups of boys, 17 with JIA and 15 healthy boys (Additional Table 5). The difference between boys with JIA and healthy boys remained very similar to the analysis in both genders. We also made a principal component analysis (PCA) -plot in the 51 children with JIA at inclusion and observed no clear gender-based groupings (Additional Fig. 1). The results of the entire Olink panel are presented in Additional Table 6.

**Table 2:** Changes in normal protein expression (NPX)-levels in 14 of 92 inflammatory proteins/cytokines in paired samples during medical treatment in 18 children with juvenile idiopathic arthritis (JIA)

	NPX levels at inclusion^a^	NPX levels at follow up^a^	*P*-values *	Increase ↑ Decrease ↓
IL-6	5.3 (4.6 – 5.9)	3.2 (2.9 – 3.6)	< 0.001	↓
IL-20	1.0 (0.9 – 1.1)	0.9 (0.8 – 0.9)	0.008	↓
TNFSF14	4.9 (4.4 – 5.3)	4.3 (4.0 – 4.6)	0.008	↓
S100A12	4.3 (3.6 – 4.9)	3.1 (2.8 – 3.3)	0.008	↓
LIF	0.9 (0.8 – 1.0)	0.8 (0.7 – 0.9)	0.02	↓
CCL19	10.3 (9.9 – 10.8)	9.8 (9.6 – 10.1)	0.02	↓
NRTN	1.0 (1.0 – 1.2)	0.9 (0.8 – 1.0)	0.03	↓
TRANCE	5.8 (5.4 – 6.2)	6.3 (6.0 – 6.5)	0.008	↑
TRAIL	8.5 (8.3 – 8.7)	8.7 (8.6 – 8.9)	0.008	↑
TNFB	5.8 (5.6 – 6.0)	7.0 (6.4 – 7.5)	0.008	↑
CX3CL1	6.0 (5.8 – 6.2)	6.3 (6.1 – 6.4)	0.008	↑
Flt3L	9.1 (9.0 – 9.3)	9.5 (9.3 – 9.7)	0.01	↑
DNER	9.6 (9.5 – 9.8)	9.8 (9.7 – 9.9)	0.02	↑
LIF-R	4.3 (4.2 – 4.4)	4.5 (4.4 – 4.6)	0.03	↑

^*^Paired T-test

^a^ NPX levels analyzed by Olink (Uppsala, Sweden) adjusted for multiple analyses using Benjamini–Hochberg method, presented as mean and confidence interval

**Table 3:** Correlations between normal protein expression (NPX)-levels in six of 92 inflammatory proteins/cytokines and pain visual analogue scale (VAS 0–10 cm) in 50 children with juvenile idiopathic arthritis (JIA) in an active disease state adjusted for age and gender

	**Correlation** **coefficients**	*P*-value*	Adjusted *p*-value**
IL-18R1	0.37	0.010	0.529
TGFβ	0.34	0.018	0.529
GDNF	0.33	0.021	0.529
FGF21	0.30	0.038	0.529
VEGFA	0.30	0.040	0.529
S100A12	0.28	0.052	0.529

^*^Spearman rank order correlation

^**^Adjusted for multiple testing (Benjamini-Hochberg)

**Table 4:** Correlations between normal protein expression (NPX)-levels in 25 of 92 inflammatory proteins versus JADAS 27 scores (0–57) in 50 children (missing data in one child) with juvenile idiopathic arthritis (JIA) in an active disease state adjusted for age and gender

Protein	Correlation coefficients	*P*-value*	Adjusted *p*-value**
S100A12	0.53	< 0.001	0.005
MMP-1	0.53	< 0.001	0.005
IL6	0.52	< 0.001	0.005
TNFSF14	0.48	< 0.001	0.015
LIF	0.44	0.002	0.029
MCP3	0.41	0.004	0.053
CX3CL1	-0.39	0.006	0.075
IL8	0.38	0.007	0.086
SIRT-2	0.37	0.009	0.089
VEGFA	0.37	0.010	0.089
CSF1	0.36	0.012	0.097
IL18R1	0.35	0.014	0.110
PD-L1	0.34	0.017	0.121
CXCL11	0.34	0.018	0.121
CCL19	0.33	0.022	0.128
IL33	0.33	0.022	0.128
TGFβ	0.32	0.025	0.131
CDCP1	0.32	0.027	0.131
DNER	-0.32	0.027	0.131
ST1A1	0.31	0.032	0.145
CASP8	0.30	0.035	0.154
FGF21	0.30	0.037	0.155
SCF	-0.30	0.040	0.159
CXCL1	0.30	0.042	0.160
CCL3	0.29	0.048	0.177

^*^Spearman rank order correlation

^**^Adjusted for multiple testing (Benjamini-Hochberg)

The 14 most significant differences in plasma levels of proteins in paired samples from 18 children with JIA are shown in Table 2. For example, NPX values of IL-6, IL-20, and TNFSF14 decreased during medical treatment, while levels of TRANCE, TNFB and TRAIL increased significantly.

The correlations between NPX values of proteins and degree of pain (VAS) in 50 (missing in one) children with JIA, adjusted for age and gender before and after adjustment for multiple testing, are presented in Table 3. Proteins IL-18R1, TGFβ, GDNF, FGF21 and VEGFA correlated significantly with degree of pain before adjustment for multiple testing. Correlations between NPX values and JADAS27, adjusted for age and gender are presented in Table 4. The S100A12, matrix metalloproteinase-1 (MMP-1), IL-6, TNFSF14 and LIF correlated significantly with JADAS27 also after adjustment for multiple testing. The NPX value of GDNF (adjusted for age and gender) did not correlate significantly with JADAS27, correlation coefficient 0.18, adjusted p-value 0.38 from multiple testing.

All results of analyses in tables and figures are presented with corrections made for multiple testing, except the analyses of protein levels in relation to degree of pain, where we have chosen to also present unadjusted results.

## Discussion

Plasma levels of 19 of 92 tested proteins involved in biological processes related to inflammation were higher in 51 JIA patients at diagnosis, in a high disease activity state, than in controls. Following treatment, a decrease in levels of inflammatory proteins was observed in the 18 participants with follow-up samples. The JADAS27 reflecting disease activity correlated with several proteins. Five proteins correlated in unadjusted analyses with pain assessed on VAS (GDNF, TGFβ, IL-18R1, FGF21 and VEGFA). Levels of GDNF, a protein involved in pain processes, correlated significantly with pain VAS scores but not with JADAS27, after adjustment for age and gender.

The well-known inflammatory proteins involved in JIA, IL-6 and S100A12, were observed at higher levels in plasma samples from patients compared with controls and decreased during treatment. Plasma IL-6 levels correlated significantly with disease activity (JADAS27), findings in line with earlier results [2]. IL-6 is also the target of one of the medications used for treatment of JIA [15]. Other proteins significantly higher in plasma from children with JIA than in controls were monocyte chemotactic protein-3 (MCP-3), a protein known to mobilize monocytes involved in the inflammatory response [16], as well as oncostatin M (OSM), which has a potentially pro-inflammatory role in arthritis [17], and hepatocyte growth factor (HGF), involved in angiogenesis and tissue regeneration [18].

Levels of the cytokine IL-20, with the capacity to increase the proliferation of synovial cells and promote neutrophil chemotaxis [19] were lower in low than high disease activity, as were those of the pro-inflammatory cytokine TNFSF14, involved in T-cell homing [20]. The cytokines TRANCE, TRAIL, and TNFB increased during treatment in our study. These cytokines are all related to the TNF cytokine family and according to the manufacturers, the TNFB results could be explained by the assay measuring both unbound TNF and TNF in complex with medical agents. This has been shown in a study on rheumatoid arthritis (RA) as well [21]. The children with JIA in this study were mainly treated with a TNF-inhibitor and a folate antagonist. Modifications by methotrexate on cytokine profiles are still unclear [22] and cytokines are also to some extent dependent on each other.

As late as 18 years after onset, many patients with JIA have an ongoing active disease with high burden of medication [23]. Pain in JIA is associated with fatigue leading to functional difficulties [6, 24, 25]. Pain and fatigue decrease a child’s ability to participate in activities like social life, school, and sports [26–28]. Even a minimal pain reduction improves the quality of life in these children [29]. Earlier research has shown that median pressure point threshold, reflecting pain tolerance, is lower in children with JIA than in controls, also in areas of the body not close to affected joints [30, 31]. This knowledge supports an altered pain perception, indicating a damage in the peripheral nerve system, a sensitization caused by the disease process. In one study, early pain severity was associated with a more severe disease outcome 15–18 years after onset, highlighting the importance of pain in disease process [32].

Many proteins involved in the inflammatory process are recognized as important mediators of inflammatory pain [33], including proteins originating from neurons [34]. It has become apparent that there is an interaction between the immune and nervous system in steady state as well as under pathological circumstances. IL-6 is involved in inflammation as well as in the pain process [15, 35]. In our study, IL-6 correlated significantly with inflammation but not with degree of pain. Other studies have shown that IL-6 is one of the pro-inflammatory cytokines, released by stimulation of TNFα, triggering the release of the final inflammatory mediator prostaglandin E2 (PGE2) and sympathetic amines that directly sensitize the nociceptors [36]. In studies of mice, IL-6 has been shown to be directly involved in the pain process of inflammatory arthritis [37].

Interestingly, plasma GDNF levels were higher in children with JIA than controls, also after adjustment for age and gender and correlated with pain in unadjusted analyses, but not with disease activity. GDNF is found in both the peripheral and the central nervous systems, but is present mainly in the peripheral neurons, particularly in the dorsal root ganglion, and in spinal dorsal horn neurons [34]. Many cell types synthesize and secrete GDNF, including glial cells, astrocytes and oligodendrocytes, Schwann cells, motor neurons, and skeletal muscle cells [38]. GDNF is a neurotrophic factor and has been found to have a neuroprotective function in the brain, protecting dopaminergic neurons, but lately it has been found to take part in nociception and pain [34]. GDNF has been shown to have an anti-nociceptive modulating role in neuropathic pain, but in experimental models of inflammatory pain it has been seen to have a pro-nociceptive effect, which could explain our findings [34, 39].

In addition to GDNF, levels of TGFβ and IL-18R1 correlated with pain. Fibroblast-like synoviocytes from patients with JIA have presented with a dysregulated TGFβ signaling compared with controls [40]. TGFβ in plasma and cerebrospinal fluid has been shown to be a biological indicator of chronic pain in patients with osteoarthritis [41] and the pro-inflammatory protein IL18 has been shown to be associated with disease activity in RA and JIA but not specifically related to degree of pain [42, 43]. Our results relate to the receptor for IL18, IL18R1, which is essential for IL18-mediated signal transduction.

The study cohort was small and drawing of conclusions must be cautious. One must consider the difficulty in interpretation of multiple cytokines in such a heterogeneous disease. A low NPX value corresponds to a lower relative concentration of a biomarker, but the NPX value of each protein is dependent on levels of all the other proteins in the analysis [12]. When comparing plasma protein levels between groups as well as in correlation analyses between NPX-levels and pain VAS results, we corrected for age and gender. We found no obvious influence on the comparisons of either of them but this should be explored in a larger cohort.

We could only find one other study that has explored a wide panel of pain-associated proteins in JIA [11]. Since the method used and the proteins studied are different, we cannot make any comparisons, but both studies support that non-inflammatory mechanisms may be involved in the pain process of those children. Our results on several cytokines involved in inflammation in JIA are in line with earlier studies. This is supportive for our results on cytokines in relation to pain and we aim to perform more, larger studies to further evaluate our findings.

## Conclusions

In this explorative study of plasma proteomics analyses related to inflammation and pain in JIA, we found that levels of proteins differed between children with JIA and controls, and that levels of some proteins also correlated with degree of pain, irrespective of age or gender. Levels of GDNF, originating from neural cells, correlated with degree of pain but not degree of inflammation. In the search for a pain-related biomarker in JIA that is not primarily explained by inflammation, GDNF could be a possible candidate for further studies.

## Supplementary Information

**Additional file 1: Additional Table 1.** Clinical and conventional laboratory variables in children with JIA.

**Additional file 2: Additional Table 2.** Levels of pain and disease activity in the children with JIA included in the study (missing data in one), presented by category of disease.

**Additional file 3: Additional Table 3.** Comparison of normal protein expression (NPX)-levels in 19 of 92 inflammatory proteins/cytokines between untreated children with juvenile idiopathic arthritis (JIA) (*n* = 51) and healthy controls (*n* = 18).

**Additional file 4: Additional Table 4.** Comparison of normal protein expression (NPX)-levels in six of 92 inflammatory proteins/cytokines between untreated children with juvenile idiopathic arthritis (JIA) (*n* = 51) and healthy controls (*n* = 18) with adjustment for age.

**Additional file 5: Additional Table 5.** Comparison of normal protein expression (NPX)-levels in six of 92 inflammatory proteins/cytokines between untreated boys with juvenile idiopathic arthritis (JIA) (*n* = 17) and healthy boys (*n* = 15) with adjustment for age.

**Additional file 6: Additional Table 6.** Olink data Welch two sample T-test healthy controls and high disease activity.

**Additional file 7: Additional Figure. 1.** A principal component analysis (PCA) plot presenting the sample-wise distribution of NPX values in 34 girls and 17 boys in a high inflammatory state of juvenile idiopathic arthritis.

## Data Availability

One main dataset is presented in Additional Table 6. Other datasets used and/or analysed during the current study are available from the corresponding author on reasonable request.

## Abbreviations

GDNF: Glial cell-line derived neurotrophic factor
HGF: Hepatocyte growth factor
IL: Interleukin
IL18R1: Interleukin 18 receptor 1
IQR: Interquartile range
JADAS27: Juvenile arthritis disease activity score 27 joints
JIA: Juvenile idiopathic arthritis
Md: Median
MMP-1: Matrix metalloproteinase 1
NPX: Normal protein expression
OSM: Oncostatin M
PCA: Principal component analysis
RA: Rheumatoid arthritis
S100A12: S100 protein A12
TGFβ: Transforming growth factor beta
TNFB: Tumor necrosis factor B
TNFSF14: Tumor necrosis factor super family member 14
TNFα: Tumor necrosis factor alpha
TRAIL: TNF related apoptosis inducing ligand
TRANCE: Tumor necrosis factor related activation-induced cytokine
VAS: Visual analogue scale
