# Supplementary material for: An explorative study on proteomic analyses related to inflammation and pain in children with juvenile idiopathic arthritis
Source: BMC Pediatr. 2023 Jul 15;23:365. doi: 10.1186/s12887-023-04181-0 (PMC10349407; doi:10.1186/s12887-023-04181-0)
Supplement: Supplementary file 1 — Additional file 1: Additional Table 1. Clinical and conventional laboratory variables in children with JIA. [file 12887_2023_4181_MOESM1_ESM.docx]

**Additional Table 1. Clinical and conventional laboratory variables in children with JIA.**

|  | Children with JIA, n = 51 |  | | Subgroup, n = 18 | | |
| --- | --- | --- | --- | --- | --- | --- |
| Variable | Total cohort at inclusion |  | At inclusion | | At follow-up | p-value* |
| Morning stiffness (min) | 15.0 (0–75) |  | 30.0 (2.5–135.0) (n = 17) | | 0.0 (0.0–6.3) | 0.009 |
| Active joints, n | 2.0 (1.0–3.0) |  | 2.0 (1.0–4.3) | | 0.0 (0.0–0.0) | < 0.001 |
| E-SR, mm | 19.0 (7.0–32.0) |  | 24.0 (9.5–41.5) | | 7.5 (2.0–10.8) | < 0.001 |
| CRP, mg/L | 2.2 (0.9–12.8) |  | 4.1 (2.2–26.0) (n = 14) | | 0.6 (0.3–0.8) (n = 10) | 0.018 |
| Leukocytes 10^9^/L | 7.0 (6.2–8.4) |  | 7.6 (6.3–9.0) | | 6.5 (5.4–7.7) | 0.004 |
| Neutrophils 10^9^/L | 3.5 (2.9–5.1) |  | 4.2 (3.0–5.4) | | 3.3 (2.1–4.4) (n = 16) | 0.004 |
| Pain VAS (0–10 cm) | 4.1 (2.2–6.3) |  | 3.9 (2.2–7.1) | | 0.3 (0.1–2.2) | 0.006 |
| GAP VAS (0–10 cm) | 3.8 (1.8–5.5) |  | 2.8 (1.4–7.4) | | 1.9 (0.2–2.9) | 0.007 |
| GAD VAS (0–10 cm) | 4.1 (2.3–6.1) |  | 4.6 (3.0–6.8) | | 0.3 (0.2–1.5) | < 0.001 |
| CHAQ score (0–3) | 0.6 (0.2–1.0) |  | 0.6 (0.1–1.3) | | 0.1 (0.0–0.4) | 0.029 |
| JADAS27 (0–27) | 9.7 (5.4–15.4) |  | 11.3 (7.4–18.9) | | 2.6 (0.3–3.7) | < 0.001 |

*related samples Wilcoxon’s signed-rank test; JIA = juvenile idiopathic arthritis; E-SR = sedimentation rate; CRP = C-reactive protein; VAS = visual analogue scale; GAP VAS = global assessment patient VAS; GAD VAS = global assessment doctor VAS; CHAQ = Child Health Assessment Questionnaire; JADAS27 = Juvenile Arthritis Disease Activity Score.
