# Supplementary material for: An explorative study on proteomic analyses related to inflammation and pain in children with juvenile idiopathic arthritis
Source: BMC Pediatr. 2023 Jul 15;23:365. doi: 10.1186/s12887-023-04181-0 (PMC10349407; doi:10.1186/s12887-023-04181-0)
Supplement: Supplementary file 2 — Additional file 2: Additional Table 2. Levels of pain and disease activity in the children with JIA included in the study (missing data in one), presented by category of disease. [file 12887_2023_4181_MOESM2_ESM.docx]

| **Additional Table 2. Levels of pain and disease activity in the children with JIA included in the study (missing data in one), presented by category of disease.** | | |
| --- | --- | --- |
| Children with JIA at inclusion, n = 51 | | |
| ILAR category | Pain VAS (0–10 cm)  Md (IQR)  (Numbers in last two rows) | JADAS27 (0–57)  Md (IQR) |
| Oligoarticular persistent (n = 25) | 2.9 (1.1–5.6) | 7.5 (4.1–11.6) |
| Enthesitis-related arthritis (n = 9) | 4.8 (2.9–7.2) | 13.0 (7.4–20.1) |
| Polyarticular RF negative (n = 7) | 6.1 (4.3–7.6) | 18.3 (13.0–30.9) |
| Juvenile psoriatic arthritis (n = 4) | 4.5 (2.6–5.6) | 7.5 (5.6–10.1) |
| Oligoarticular extended (n = 3) | 3.5; 3.9; 7.7 | 9.5; 9.8; 18.1 |
| Polyarticular RF positive (n = 2) | 2.7; 8.3 | 14.2; 31.3 |
| JIA = juvenile idiopathic arthritis; ILAR = International League of Associations for Rheumatology; VAS = visual analogue scale; JADAS27 = Juvenile Arthritis Disease Activity Score; Md = median; IQR = interquartile range; RF = rheumatoid factor. | | |
