# Supplementary material for: An explorative study on proteomic analyses related to inflammation and pain in children with juvenile idiopathic arthritis
Source: BMC Pediatr. 2023 Jul 15;23:365. doi: 10.1186/s12887-023-04181-0 (PMC10349407; doi:10.1186/s12887-023-04181-0)
Supplement: Supplementary file 3 — Additional file 3: Additional Table 3. Comparison of normal protein expression (NPX)-levels in 19 of 92 inflammatory proteins/cytokines between untreated children with juvenile idiopathic arthritis (JIA) (n = 51) and healthy controls (n = 18). [file 12887_2023_4181_MOESM3_ESM.docx]

**Additional table 3. Comparison of normal protein expression (NPX)-levels in 19 of 92 inflammatory proteins/cytokines between untreated children with juvenile idiopathic arthritis (JIA) (n=51) and healthy controls (n=18).**

| Protein |  | NPX-level | |  |  | |  | Confidence interval | |  |
| --- | --- | --- | --- | --- | --- | --- | --- | --- | --- | --- |
|  |  | **JIA n=51**  **Mean (SD)** | **Control n=18**  **Mean (SD)** | **Mean difference** | | **p-value*** |  | **Low** | **High** |  |
| MCP-3 |  | 2.7 (0.9) | 2.1 (0.4) | - 0.7 | | < 0.001 |  | - 1.0 | - 0.4 |  |
| GDNFα |  | 2.8 (0.4) | 2.3 (0.3) | - 0.5 | | < 0.001 |  | - 0.7 | - 0.3 |  |
| IL-6 |  | 4.8 (1.8) | 3.2 (0.8) | - 1.6 | | < 0.001 |  | - 2.2 | - 1.0 |  |
| OSM |  | 4.6 (0.9) | 3.7 (0.6) | - 0.9 | | < 0.001 |  | - 1.3 | - 0.5 |  |
| HGF |  | 9.0 (0.4) | 8.6 (0.2) | - 0.5 | | < 0.001 |  | - 0.6 | - 0.3 |  |
| S100A12  CSF-1  FGF-19  VEGFA  NT-3  CXCL10  FGF-21  PD-L1  TNFSF14  DNER  ARTN  MMP-1  TNFRSF9  CCL23 |  | 3.7 (1.4)  10.4 (0.2)  8.3 (0.9)  11.0 (0.5)  3.7 (0.7)  9.8 (1.0)  4.3 (1.7)  6.9 (0.3)  4.8 (0.8)  9.7 (0.3)  1.3 (0.4)  10.6 (1.6)  8.2 (0.6)  10.3 (0.6) | 2.6 (0.3)  10.3 (0.2)  7.5 (0.7)  10.6 (0.3)  3.2 (0.4)  9.0 (0.7)  5.5 (1.2)  6.6 (0.3)  4.3 (0.4)  9.9 (0.2)  1.0 (0.3)  9.6 (1.1)  8.6 (0.4)  9.9 (0.5) | - 1.0  - 0.3  - 0.8  - 0.4  - 0.5  - 0.8  1.2  - 0.3  - 0.5  0.2  - 0.3  - 1.0  0.4  - 0.4 | | < 0.001  < 0.001  0.007  0.007  0.02  0.02  0.02  0.02  0.02  0.02  0.02  0.03  0.04  0.04 |  | - 1.5  - 0.4  -1.2  - 0.6  - 0.8  - 1.2  0.4  - 0.5  - 0.8  0.1  - 0.4  - 1.7  0.1  - 0.7 | - 0.6  - 0.2  - 0.4  - 0.2  - 0.2  - 0.3  2.0  - 0.1  - 0.2  0.3  - 0.1  - 0.3  0.6  - 0.1 |  |

*Independent samples T test; MCP-3= Monocyte Chemotactic Protein 3; GDNFα = Glial Derived Neurotrophic Factor α; IL-6 = Interleukin 6; OSM = Oncostatin M; HGF = Hepatocyte Growth Factor; S100A12 = S100 Calcium Binding Protein A12.
