# Supplementary material for: An explorative study on proteomic analyses related to inflammation and pain in children with juvenile idiopathic arthritis
Source: BMC Pediatr. 2023 Jul 15;23:365. doi: 10.1186/s12887-023-04181-0 (PMC10349407; doi:10.1186/s12887-023-04181-0)
Supplement: Supplementary file 4 — Additional file 4: Additional Table 4. Comparison of normal protein expression (NPX)-levels in six of 92 inflammatory proteins/cytokines between untreated children with juvenile idiopathic arthritis (JIA) (n = 51) and healthy controls (n = 18) with adjustment for age. [file 12887_2023_4181_MOESM4_ESM.docx]

| Additional Table 4. Comparison of normal protein expression (NPX)-levels in six of 92 inflammatory proteins/cytokines between untreated children with juvenile idiopathic arthritis (JIA) (n = 51) and healthy controls (n = 18) with adjustment for age | | | | | | | | | | |  |
| --- | --- | --- | --- | --- | --- | --- | --- | --- | --- | --- | --- |
| Protein |  | **NPX level** | |  | **Crude** | |  | | **Adjusted for age** | | |
|  |  | **JIA n=51**  **Mean (SD)*** | **Control n=18**  **Mean(SD)*** |  | **Mean** | **p- value**** |  | **Mean** | | **p-value**** |  |
| MCP-3 |  | 2.7 (0.9) | 2.1 (0.4) |  | 0.66 | < 0.001 |  | 0.90 | | < 0.001 |  |
| GDNFα |  | 2.8 (0.4) | 2.3 (0.3) |  | 0.48 | < 0.001 |  | 0.38 | | < 0.001 |  |
| IL-6 |  | 4.8 (1.8) | 3.2 (0.8) |  | 1.6 | < 0.001 |  | 1.77 | | < 0.001 |  |
| OSM |  | 4.6 (0.9) | 3.7 (0.6) |  | 0.9 | < 0.001 |  | 0.9 | | < 0.001 |  |
| HGF |  | 9.0 (0.4) | 8.6 (0.2) |  | 0.4 | < 0.001 |  | 0.5 | | < 0.001 |  |
| S100A12 |  | 3.7 (1.4) | 2.6 (0.3) |  | 1.0 | < 0.001 |  | 1.4 | | < 0.001 |  |

*Independent samples T test; **Linear regression; MCP-3= Monocyte Chemotactic Protein 3; GDNFα = Glial Derived Neurotrophic Factor α; IL-6 = Interleukin 6; OSM = Oncostatin M; HGF = Hepatocyte Growth Factor; S100A12 = S100 Calcium Binding Protein A12.
