# Supplementary material for: An explorative study on proteomic analyses related to inflammation and pain in children with juvenile idiopathic arthritis
Source: BMC Pediatr. 2023 Jul 15;23:365. doi: 10.1186/s12887-023-04181-0 (PMC10349407; doi:10.1186/s12887-023-04181-0)
Supplement: Supplementary file 5 — Additional file 5: Additional Table 5. Comparison of normal protein expression (NPX)-levels in six of 92 inflammatory proteins/cytokines between untreated boys with juvenile idiopathic arthritis (JIA) (n = 17) and healthy boys (n = 15) with adjustment for age. [file 12887_2023_4181_MOESM5_ESM.docx]

| Additional Table 5. Comparison of normal protein expression (NPX)-levels in six of 92 inflammatory proteins/cytokines between untreated boys with juvenile idiopathic arthritis (JIA) (n = 17) and healthy boys (n = 15) with adjustment for age | | | | | | | | | | |  |
| --- | --- | --- | --- | --- | --- | --- | --- | --- | --- | --- | --- |
| Protein |  | **NPX level** | |  | **Crude** | |  | | **Adjusted for age** | | |
|  |  | **JIA n=17**  **Mean (SD)*** | **Control n=15**  **Mean (SD)*** |  | **Mean** | **p- value**** |  | **Mean** | | **p-value**** |  |
| MCP-3 |  | 2.7 (0.7) | 2.1 (0.4) |  | 0.6 | 0.004 |  | 0.7 | | 0.006 |  |
| GDNFα |  | 2.9 (0.4) | 2.3 (0.3) |  | 0.6 | < 0.001 |  | 0.5 | | 0.004 |  |
| IL-6 |  | 4.8 (1.9) | 3.3 (0.8) |  | 1.5 | 0.006 |  | 1.2 | | 0.039 |  |
| OSM |  | 4.6 (1.0) | 3.7 (0.7) |  | 0.9 | 0.006 |  | 0.8 | | 0.028 |  |
| HGF |  | 9.1 (0.5) | 8.6 (0.3) |  | 0.5 | < 0.001 |  | 0.5 | | 0.004 |  |
| S100A12 |  | 3.8 (1.6) | 2.7 (0.3) |  | 1.1 | 0.014 |  | 0.9 | | 0.053 |  |

*Independent samples T test; **Linear regression; MCP-3= Monocyte Chemotactic Protein 3; GDNFα = Glial Derived Neurotrophic Factor α; IL-6 = Interleukin 6; OSM = Oncostatin M; HGF = Hepatocyte Growth Factor; S100A12 = S100 Calcium Binding Protein A12.
