# Supplementary material for: An explorative study on proteomic analyses related to inflammation and pain in children with juvenile idiopathic arthritis
Source: BMC Pediatr. 2023 Jul 15;23:365. doi: 10.1186/s12887-023-04181-0 (PMC10349407; doi:10.1186/s12887-023-04181-0)
Supplement: Supplementary file 6 — Additional file 6: Additional Table 6. Olink data Welch two sample T-test healthy controls and high disease activity. [file 12887_2023_4181_MOESM6_ESM.pdf]

Additional Table 6. Olink data Welch two sample T-test healthy controls and high disease activity

Name	missing freq	estimate	control	High disease activity	statistic	p-value	parameter	conf.low	conf.high	adj p-value	Threshold
HGF	0	-0,46	8,59	9,04	-5,59	1,05E-06	48,29	-0,62	-0,29	8,73E-05	Significant
IL6	0	-1,62	3,18	4,80	-5,25	1,90E-06	63,55	-2,24	-1,01	8,73E-05	Significant
EN-RAGE	0	-1,04	2,65	3,69	-4,90	6,99E-06	63,13	-1,47	-0,62	2,01E-04	Significant
GDNF	0	-0,48	2,29	2,77	-5,10	8,74E-06	39,89	-0,66	-0,29	2,01E-04	Significant
OSM	0	-0,93	3,66	4,59	-4,73	2,82E-05	39,93	-1,32	-0,53	5,19E-04	Significant
MCP-3	0	-0,66	2,08	2,74	-4,36	4,74E-05	65,50	-0,96	-0,36	6,46E-04	Significant
CSF-1	0	-0,29	10,08	10,37	-4,67	4,92E-05	32,65	-0,42	-0,17	6,46E-04	Significant
FGF-19	0	-0,80	7,52	8,32	-3,73	6,18E-04	38,96	-1,24	-0,37	6,92E-03	Significant
VEGFA	0	-0,40	10,64	11,04	-3,63	6,77E-04	48,93	-0,62	-0,18	6,92E-03	Significant
NT-3	0	-0,47	3,24	3,71	-3,30	1,81E-03	48,41	-0,76	-0,18	1,67E-02	Significant
CXCL10	0	-0,75	9,04	9,79	-3,29	2,00E-03	43,25	-1,21	-0,29	1,67E-02	Significant
FGF-21	0	1,18	5,52	4,34	3,11	3,38E-03	41,49	0,41	1,95	2,43E-02	Significant
PD-L1	0	-0,29	6,61	6,90	-3,18	3,48E-03	29,31	-0,48	-0,10	2,43E-02	Significant
TNFSF14	0	-0,46	4,29	4,75	-3,02	3,79E-03	58,07	-0,77	-0,16	2,43E-02	Significant
DNER	0	0,19	9,85	9,66	3,05	4,17E-03	37,45	0,06	0,31	2,43E-02	Significant
ARTN	0.47778	-0,26	1,00	1,26	-3,00	4,23E-03	50,70	-0,44	-0,09	2,43E-02	Significant
MMP-1	0	-1,01	9,62	10,62	-2,87	6,45E-03	41,36	-1,72	-0,30	3,49E-02	Significant
TNFRSF9	0	0,37	8,61	8,25	2,81	7,85E-03	37,20	0,10	0,63	4,01E-02	Significant
CCL23	0	-0,38	9,89	10,27	-2,79	8,35E-03	36,83	-0,65	-0,10	4,04E-02	Significant
FGF-23	0	-0,24	3,07	3,31	-2,65	1,09E-02	49,12	-0,42	-0,06	5,03E-02	Non-significant
IL13	0.57778	-0,41	1,16	1,57	-2,29	2,52E-02	64,18	-0,77	-0,05	1,10E-01	Non-significant
Beta-NGF	0.55556	-0,07	1,21	1,27	-2,29	2,72E-02	41,16	-0,12	-0,01	1,14E-01	Non-significant
CCL4	0	-0,35	6,00	6,35	-2,24	3,23E-02	30,93	-0,67	-0,03	1,29E-01	Non-significant
CDCP1	0	-0,30	3,02	3,32	-2,18	3,65E-02	34,77	-0,58	-0,02	1,40E-01	Non-significant
CCL25	0	0,34	6,57	6,23	2,17	3,90E-02	27,08	0,02	0,67	1,43E-01	Non-significant
CCL28	0	-0,20	2,27	2,47	-2,10	4,16E-02	46,21	-0,39	-0,01	1,46E-01	Non-significant
IL-20RA	0.15556	-0,17	1,49	1,66	-2,05	4,45E-02	60,12	-0,33	0,00	1,46E-01	Non-significant
Flt3L	0	-0,23	8,96	9,19	-2,08	4,45E-02	34,68	-0,46	-0,01	1,46E-01	Non-significant
CD244	0	-0,18	7,64	7,82	-2,00	5,40E-02	32,65	-0,37	0,00	1,71E-01	Non-significant
CCL3	0	-0,32	4,62	4,94	-1,95	5,70E-02	49,04	-0,65	0,01	1,75E-01	Non-significant

IL-18R1	0	-0,22	8,70	8,92	-1,91	6,52E-02	30,83	-0,45	0,01	1,90E-01	Non-significant
TRANCE	0	-0,36	5,67	6,03	-1,90	6,59E-02	33,13	-0,75	0,03	1,90E-01	Non-significant
IL33	0.72222	0,23	1,68	1,46	1,88	7,48E-02	19,34	-0,02	0,48	2,05E-01	Non-significant
IL-17A	0.01111	-0,27	2,07	2,35	-1,83	7,58E-02	33,35	-0,57	0,03	2,05E-01	Non-significant
TNFB	0	0,28	6,22	5,95	1,80	8,08E-02	32,13	-0,04	0,59	2,12E-01	Non-significant
IL7	0	-0,27	3,16	3,43	-1,71	9,47E-02	47,27	-0,60	0,05	2,42E-01	Non-significant
ADA	0	0,13	6,16	6,03	1,57	1,23E-01	43,11	-0,04	0,30	3,05E-01	Non-significant
ST1A1	0	-0,36	2,70	3,06	-1,50	1,42E-01	36,76	-0,84	0,13	3,45E-01	Non-significant
IL-24	0.07778	-0,23	1,66	1,90	-1,44	1,57E-01	36,45	-0,56	0,09	3,71E-01	Non-significant
MCP-2	0	-0,29	8,48	8,77	-1,30	2,05E-01	25,22	-0,75	0,17	4,72E-01	Non-significant
AXIN1	0.01111	-0,51	4,11	4,62	-1,25	2,20E-01	31,19	-1,33	0,32	4,93E-01	Non-significant
CD40	0	-0,16	11,65	11,81	-1,23	2,26E-01	38,52	-0,42	0,10	4,95E-01	Non-significant
4E-BP1	0	0,29	8,79	8,49	1,20	2,38E-01	30,63	-0,20	0,79	5,10E-01	Non-significant
SLAMF1	0	-0,08	1,92	1,99	-1,15	2,56E-01	50,85	-0,21	0,06	5,22E-01	Non-significant
NRTN	0.54444	-0,11	1,06	1,17	-1,13	2,64E-01	60,85	-0,31	0,09	5,22E-01	Non-significant
IL-1 alpha	0.34444	0,39	0,06	-0,32	1,13	2,70E-01	25,94	-0,32	1,09	5,22E-01	Non-significant
SIRT2	0	-0,36	4,91	5,27	-1,11	2,72E-01	39,72	-1,01	0,29	5,22E-01	Non-significant
CXCL6	0	0,25	8,71	8,46	1,12	2,73E-01	34,20	-0,21	0,71	5,22E-01	Non-significant
IL-22 RA1	0.16667	-0,24	2,47	2,71	-1,11	2,79E-01	24,08	-0,67	0,20	5,24E-01	Non-significant
MCP-1	0	0,15	11,54	11,39	1,06	2,96E-01	31,55	-0,14	0,45	5,45E-01	Non-significant
STAMBP	0	-0,25	5,92	6,17	-1,01	3,18E-01	41,74	-0,75	0,25	5,51E-01	Non-significant
TSLP	0.36667	-0,14	0,77	0,91	-1,00	3,19E-01	66,96	-0,41	0,14	5,51E-01	Non-significant
uPA	0	0,08	10,79	10,70	1,01	3,20E-01	38,76	-0,08	0,25	5,51E-01	Non-significant
CXCL1	0	0,27	8,62	8,35	1,00	3,24E-01	35,61	-0,28	0,82	5,51E-01	Non-significant
IL-15RA	0	0,10	2,08	1,98	0,98	3,38E-01	21,05	-0,11	0,31	5,57E-01	Non-significant
IL-2RB	0.24444	-0,10	1,75	1,85	-0,95	3,44E-01	66,24	-0,31	0,11	5,57E-01	Non-significant
IFN-gamma	0	-0,30	6,65	6,95	-0,96	3,45E-01	39,21	-0,94	0,34	5,57E-01	Non-significant
CCL11	0	0,14	6,66	6,52	0,93	3,60E-01	27,29	-0,17	0,44	5,71E-01	Non-significant
IL8	0	0,19	5,06	4,87	0,91	3,70E-01	29,39	-0,23	0,60	5,76E-01	Non-significant
IL-10RB	0	-0,07	6,12	6,19	-0,88	3,86E-01	29,97	-0,24	0,09	5,76E-01	Non-significant
TRAIL	0	-0,06	8,49	8,56	-0,87	3,88E-01	36,91	-0,20	0,08	5,76E-01	Non-significant
IL4	0.12222	-0,14	1,81	1,94	-0,87	3,88E-01	30,58	-0,45	0,18	5,76E-01	Non-significant
IL2	0.97778	-0,04	1,06	1,09	-0,79	4,36E-01	39,05	-0,13	0,06	6,37E-01	Non-significant

TNF	0	-0,12	4,33	4,45	-0,75	4,57E-01	29,35	-0,46	0,21	6,54E-01	Non-significant
FGF-5	0.01111	-0,07	1,48	1,55	-0,74	4,62E-01	31,38	-0,26	0,12	6,54E-01	Non-significant
CXCL11	0	-0,26	8,23	8,50	-0,73	4,69E-01	26,56	-1,00	0,47	6,54E-01	Non-significant
.P TGF-beta	0	-0,10	7,88	7,97	-0,67	5,05E-01	34,02	-0,38	0,19	6,93E-01	Non-significant
CASP-8	0	0,08	2,23	2,14	0,66	5,15E-01	31,20	-0,18	0,35	6,97E-01	Non-significant
CXCL9	0	-0,20	7,46	7,66	-0,64	5,26E-01	27,88	-0,83	0,44	7,01E-01	Non-significant
OPG	0	-0,05	10,15	10,20	-0,59	5,61E-01	33,70	-0,22	0,12	7,17E-01	Non-significant
MCP-4	0	-0,15	12,79	12,94	-0,58	5,69E-01	26,21	-0,69	0,39	7,17E-01	Non-significant
IL-20	0.33333	0,07	1,06	0,99	0,57	5,73E-01	18,27	-0,20	0,35	7,17E-01	Non-significant
CST5	0	-0,07	5,66	5,72	-0,56	5,77E-01	48,15	-0,31	0,18	7,17E-01	Non-significant
SCF	0	0,06	9,59	9,53	0,56	5,81E-01	36,64	-0,15	0,27	7,17E-01	Non-significant
IL-12B	0	-0,08	6,95	7,03	-0,55	5,89E-01	28,09	-0,39	0,23	7,17E-01	Non-significant
CCL20	0	0,10	7,89	7,79	0,53	5,97E-01	38,81	-0,28	0,47	7,17E-01	Non-significant
TWEAK	0	-0,04	10,08	10,12	-0,53	6,01E-01	48,00	-0,20	0,11	7,17E-01	Non-significant
CCL19	0	-0,10	10,06	10,16	-0,45	6,53E-01	28,66	-0,57	0,36	7,61E-01	Non-significant
CD6	0	-0,07	6,40	6,47	-0,45	6,53E-01	30,47	-0,39	0,25	7,61E-01	Non-significant
LIF	0.31111	-0,07	0,99	1,06	-0,40	6,92E-01	39,27	-0,42	0,28	7,88E-01	Non-significant
CXCL5	0	-0,15	10,18	10,33	-0,40	6,94E-01	34,27	-0,94	0,63	7,88E-01	Non-significant
IL-17C	0.13333	-0,05	1,80	1,85	-0,36	7,21E-01	24,64	-0,32	0,23	8,00E-01	Non-significant
MMP-10	0	-0,08	8,91	8,99	-0,35	7,28E-01	25,12	-0,53	0,37	8,00E-01	Non-significant
LIF-R	0	0,02	4,36	4,35	0,35	7,31E-01	38,49	-0,09	0,13	8,00E-01	Non-significant
CD5	0	-0,04	6,01	6,05	-0,33	7,44E-01	28,34	-0,31	0,23	8,05E-01	Non-significant
IL10	0	-0,07	4,76	4,83	-0,30	7,64E-01	25,90	-0,58	0,43	8,18E-01	Non-significant
IL-10RA	0.02222	-0,05	1,72	1,76	-0,26	7,94E-01	25,80	-0,40	0,31	8,37E-01	Non-significant
TGF-alpha	0	0,02	3,89	3,87	0,25	8,00E-01	49,78	-0,14	0,18	8,37E-01	Non-significant
IL18	0	0,02	8,89	8,87	0,15	8,84E-01	29,04	-0,28	0,32	9,13E-01	Non-significant
IL5	0.17778	0,04	1,99	1,94	0,11	9,14E-01	20,15	-0,79	0,88	9,26E-01	Non-significant
CD8A	0	0,02	11,51	11,49	0,11	9,16E-01	40,39	-0,32	0,36	9,26E-01	Non-significant
CX3CL1	0	0,00	6,11	6,11	-0,04	9,68E-01	30,73	-0,20	0,20	9,68E-01	Non-significant
