# Supplementary figures and images for: An explorative study on proteomic analyses related to inflammation and pain in children with juvenile idiopathic arthritis
Source: BMC Pediatr. 2023 Jul 15;23:365. doi: 10.1186/s12887-023-04181-0 (PMC10349407; doi:10.1186/s12887-023-04181-0)

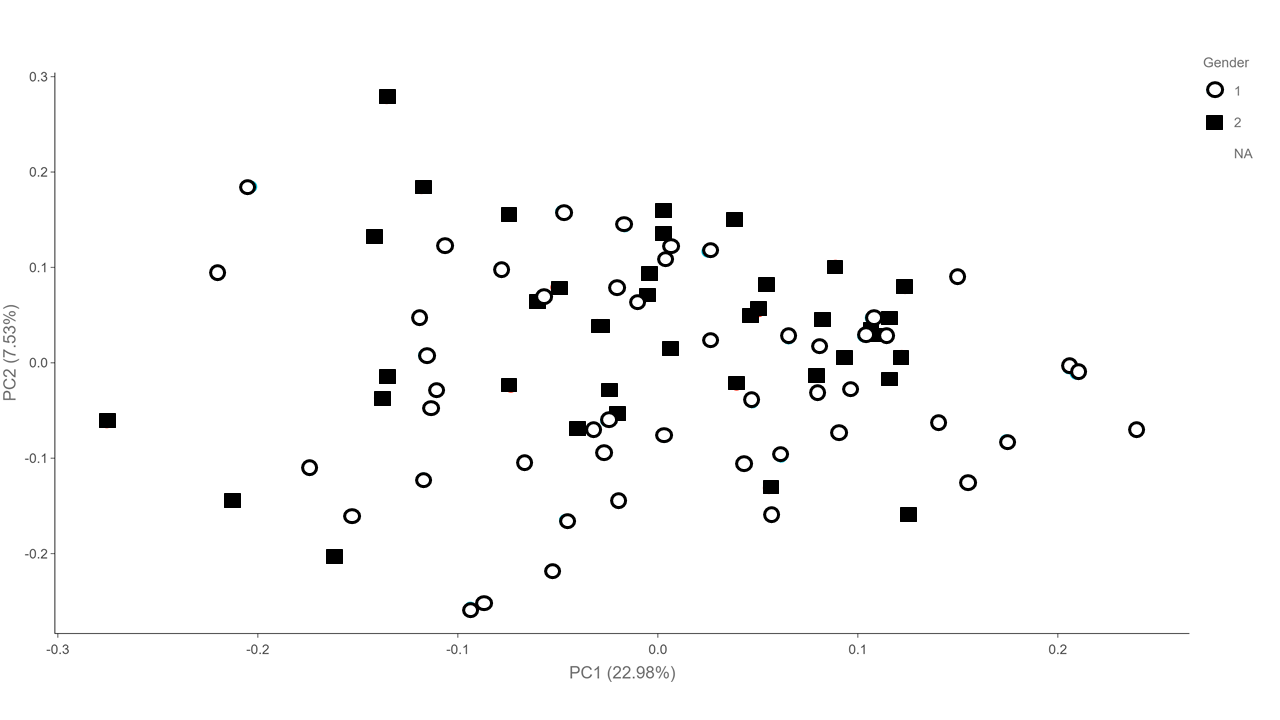

Supplement: Supplementary file 7 — Additional file 7: Additional Figure. 1. A principal component analysis (PCA) plot presenting the sample-wise distribution of NPX values in 34 girls and 17 boys in a high inflammatory state of juvenile idiopathic arthritis. [file 12887_2023_4181_MOESM7_ESM.png]
